# Supplementary material for: Benchmarking common quantification strategies for large-scale phosphoproteomics
Source: Nat Commun. 2018 Mar 13;9:1045. doi: 10.1038/s41467-018-03309-6 (PMC5849679; doi:10.1038/s41467-018-03309-6)
Supplement: Supplementary file 1 — Supplementary Information [file 41467_2018_3309_MOESM1_ESM.pdf]

“Benchmarking common quantification strategies for large-scale phosphoproteomics”

from Hogrebe *et al.*

Supplementary Figures

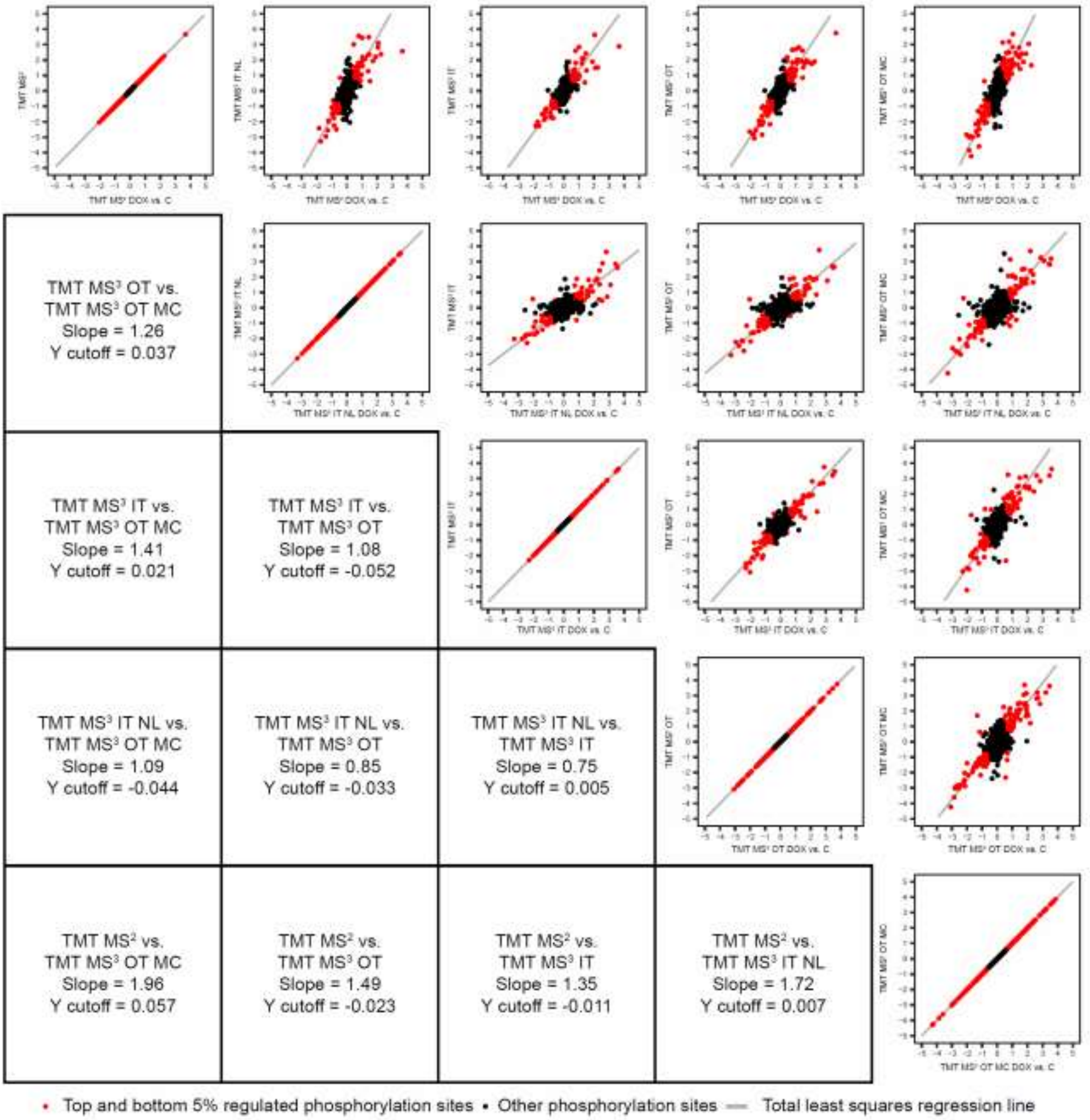

Supplementary Figure 1: Correlation of phosphorylation optimized TMT methods. Correlation plots and linear regression line characteristics for the heat map shown in Fig 1b). The 5% highest and lowest log2 ratios, which were recorded after 2h treatment of U2OS cells with Doxorubicin (DOX) vs. DMSO (C), are marked in red and were used for calculation of the linear regression lines shown in grey.

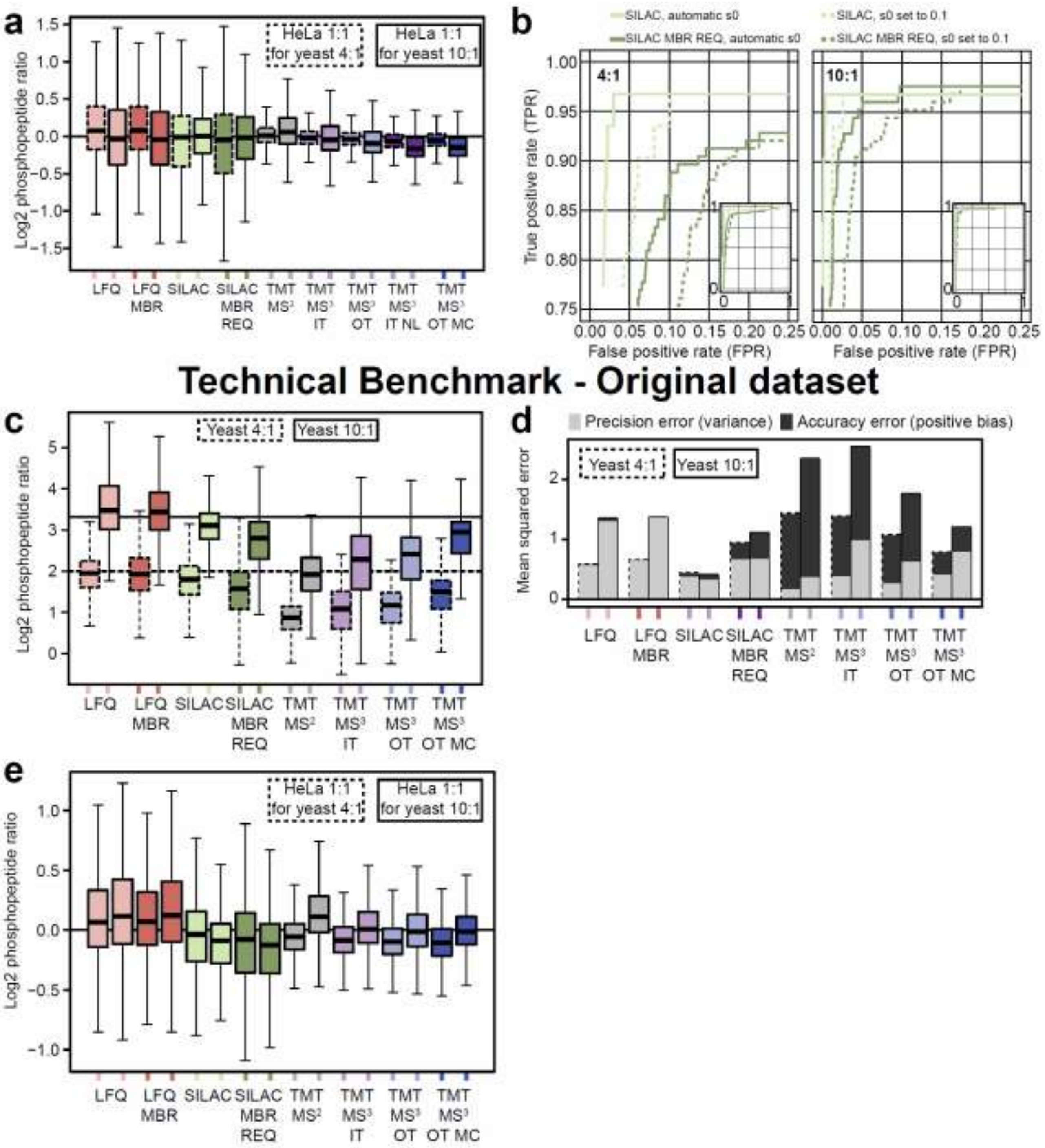
Technical Benchmark - Original dataset
a
Log2 phosphopeptide ratio
HeLa 1:1 for yeast 4:1
HeLa 1:1 for yeast 10:1
LFQ
LFQ MBR
SILAC
SILAC MBR REQ
TMT MS²
TMT MS³ IT
TMT MS³ OT
TMT MS³ IT NL
TMT MS³ OT MC
b
SILAC, automatic s0
SILAC MBR REQ, automatic s0
SILAC, s0 set to 0.1
SILAC MBR REQ, s0 set to 0.1
4:1
10:1
True positive rate (TPR)
False positive rate (FPR)
c
Yeast 4:1
Yeast 10:1
Log2 phosphopeptide ratio
d
Precision error (variance)
Accuracy error (positive bias)
Mean squared error
Yeast 4:1
Yeast 10:1
e
HeLa 1:1 for yeast 4:1
HeLa 1:1 for yeast 10:1
Log2 phosphopeptide ratio

Supplementary Figure 2: Evaluation of quantification methods for HeLa ratios. a) Box plot showing HeLa 4:1 and 10:1 phosphopeptide ratios for the different quantification methods. Boxes mark the first and third quartile, with the median highlighted as dash, and whiskers marking the minimum/maximum value within 1.5 interquartile range. Outliers are not shown. Both LFQ and SILAC were tested with and without the MaxQuant feature match-between-runs (MBR), and SILAC additionally with both MBR and requantify (REQ) activated. As SILAC-MBR only results were essentially identical to SILAC only, they are not shown here. Two HeLa 1:1 ratios are displayed individually for their respective yeast 4:1 and 10:1 channels. b) Receiver operating characteristic (ROC) curves for SILAC only were calculated by using the d-score from SAM testing as an indicator for significant regulation at 4:1 and 10:1 dilution. SAM testing for significantly regulated phosphopeptides was performed at default settings (s0 estimation automatic, solid lines) and with s0 set to 0.1. This was done to test for impact of the high s0 estimated by the SamR package for SILAC, as described in Supplementary Table 2. ROC plots are presented as zoomed-in excerpts from the total plots, shown on the bottom right each. c-e) The original data set which had to be remeasured for a) and Fig. 2, because the third LFQ 10 replicate did not contain enough yeast peptide identifications for the SAM analysis in Fig. 2 d). In this original data set, SILAC and TMT MS samples were injected without dilution, so that each labeling channel equaled one LFQ injection. c/e) Box plots showing c) yeast and e) HeLa 4:1 and 10:1 phosphopeptide ratios in the same way as in a). d) Mean squared errors were calculated as a sum of positive bias and variance for each method and all replicates.

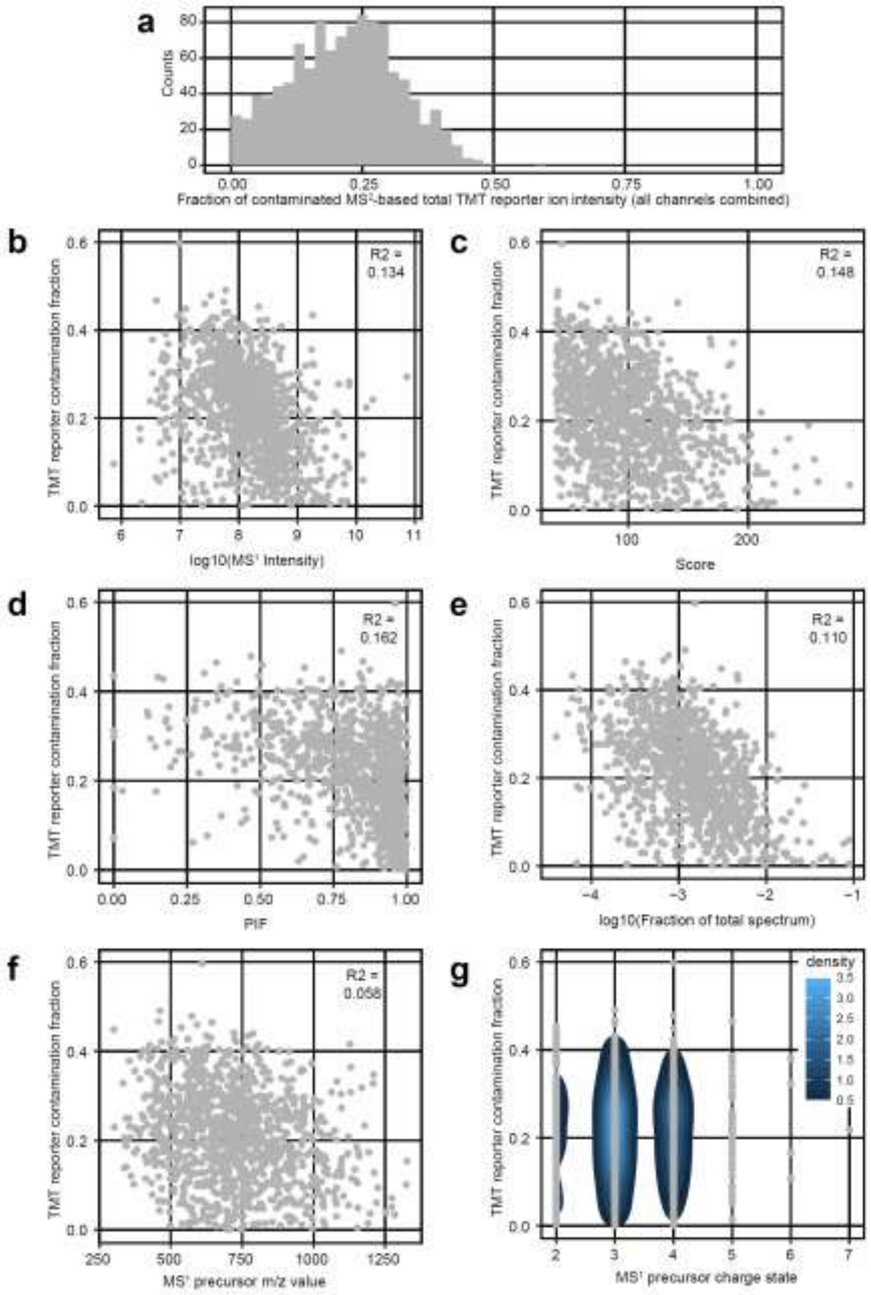

Supplementary Figure 3: MS^2 based TMT precursor ion contamination. a) Since true target ratios from the comparison in Supplementary Fig 2 b-d) were known, the degree of TMT reporter ion contamination for MS^2-measurement could be calculated by correlating target values (1, 4 and 10) vs. measured TMT reporter ion intensities. The y-axis cutoff would yield the contamination intensity, which is plotted as a histogram distribution for all features from the MaxQuant evidence-file. We found that the degree of contamination correlated to some extent with b) MS^1 precursor intensity, c) Andromeda score, d) precursor isolation fraction (PIF)-value, e) the fraction of database-identified peaks of the total MS^2 spectrum, f) the MS^1 precursor m/z value, but not g) the MS^1 precursor charge state. Interestingly, a high PIF value was a relatively poor indicator of MS^2 spectrum contamination, indicating that even very lowly abundant background peptides can significantly contribute to contamination. Density calculation was added to better visualize the distribution in g).

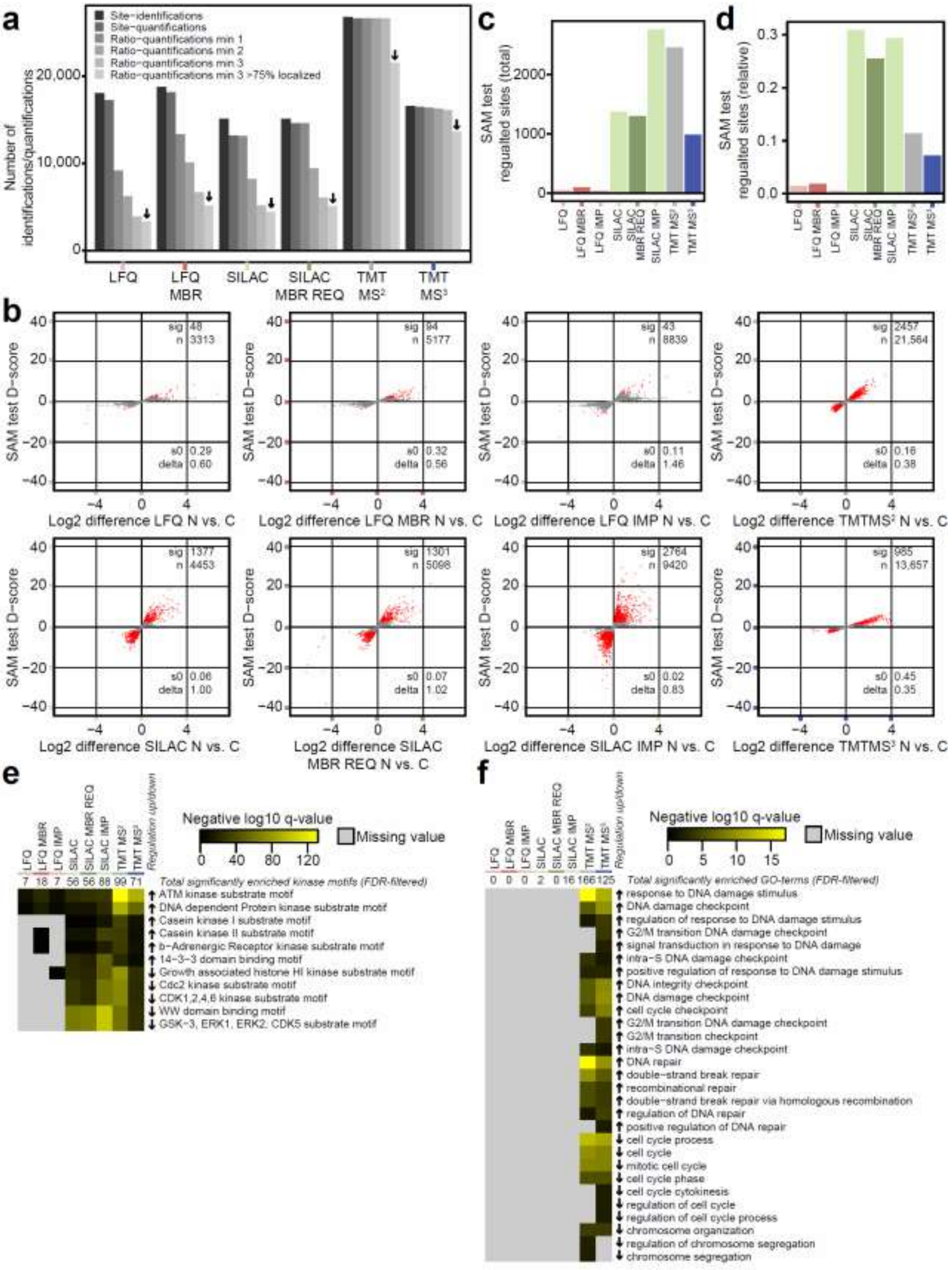
a
Site-identifications
Site-quantifications
Ratio-quantifications min 1
Ratio-quantifications min 2
Ratio-quantifications min 3
Ratio-quantifications min 3 >75% localized
Number of identifications/quantifications
20,000
10,000
0
LFQ
LFQ MBR
SILAC
SILAC MBR REQ
TMT MS²
TMT MS³
c
SAM test regualted sites (total)
2000
1000
0
LFQ
LFQ MBR
LFQ IMP
SILAC
SILAC MBR REQ
SILAC IMP
TMT MS²
TMT MS³
d
SAM test regualted sites (relative)
0.3
0.2
0.1
0.0
b
SAM test D-score
Log2 difference LFQ N vs. C
sig 48 n 3313 s0 0.29 delta 0.60
Log2 difference LFQ MBR N vs. C
sig 94 n 5177 s0 0.32 delta 0.56
Log2 difference LFQ IMP N vs. C
sig 43 n 8839 s0 0.11 delta 1.46
Log2 difference TMTMS² N vs. C
sig 2457 n 21,564 s0 0.16 delta 0.38
Log2 difference SILAC N vs. C
sig 1377 n 4453 s0 0.06 delta 1.00
Log2 difference SILAC MBR REQ N vs. C
sig 1301 n 5098 s0 0.07 delta 1.02
Log2 difference SILAC IMP N vs. C
sig 2764 n 9420 s0 0.02 delta 0.83
Log2 difference TMTMS³ N vs. C
sig 985 n 13,657 s0 0.45 delta 0.35
e
Negative log10 q-value
0 40 80 120
Missing value
Regulation up/down
7 18 7 56 56 88 99 71
Total significantly enriched kinase motifs (FDR-filtered)
ATM kinase substrate motif
DNA dependent Protein kinase substrate motif
Casein kinase I substrate motif
Casein kinase II substrate motif
b-Adrenergic Receptor kinase substrate motif
14-3-3 domain binding motif
Growth associated histone HI kinase substrate motif
Cdc2 kinase substrate motif
CDK1,2,4,6 kinase substrate motif
WW domain binding motif
GSK-3, ERK1, ERK2, CDK5 substrate motif
f
Negative log10 q-value
0 5 10 15
Missing value
0 0 0 2 0 16 166 125
Total significantly enriched GO-terms (FDR-filtered)
response to DNA damage stimulus
DNA damage checkpoint
regulation of response to DNA damage stimulus
G2/M transition DNA damage checkpoint
signal transduction in response to DNA damage
intra-S DNA damage checkpoint
positive regulation of response to DNA damage stimulus
DNA integrity checkpoint
DNA damage checkpoint
cell cycle checkpoint
G2/M transition DNA damage checkpoint
G2/M transition checkpoint
intra-S DNA damage checkpoint
DNA repair
double-strand break repair
recombinational repair
double-strand break repair via homologous recombination
regulation of DNA repair
positive regulation of DNA repair
cell cycle process
cell cycle
mitotic cell cycle
cell cycle phase
cell cycle cytokinesis
regulation of cell cycle
regulation of cell cycle process
chromosome organization
regulation of chromosome segregation
chromosome segregation

Supplementary Figure 4: Evaluation of quantification methods in a biological setting using 4NQO. a) Bar plot showing total numbers of identified and quantified phosphopeptides for all replicates of each quantification method, respectively. Calculations of ratios were performed within biological replicates and filtered for measurement in a minimum of one, two or three replicates, and >75% confident phosphorylation site localization. For further analysis, ratios quantified in all three replicates only and with a localization probability of at least 75% (black arrows) were used. b) Significance analysis of microarrays (SAM)-based identification of significantly regulated phosphorylation sites was performed with two sample unpaired t-test and standard settings (s0 estimation automatic, delta estimation based on FDR = 0.20). Significantly regulated phosphorylation sites (sig) are highlighted in red, non-significant ones in grey. Applied s0 and delta values, as well as the total number of tested phosphorylation sites (n) are shown. For LFQ and SILAC nearest neighbour imputation (IMP), phosphorylation sites quantified in at least one replicate and with a localization probability of at least 75% were used. Imputation was performed with the R package “impute” incorporated in the “samr” package using standard settings. c/d) The bar plots show the number of significantly regulated phosphorylation sites for each quantification method c) in total, and d) as a fraction relative to the total number of tested sites. e/f) Heat maps showing e) a kinase motif and f) GO-term enrichment of significantly SAM-up/down-regulated phosphorylation sites from b) vs the respective non-regulated sites as background. Enrichment was performed using Fisher exact tests within Perseus with relative enrichment on gene level and an FDR of 0.02. The numbers above the heatmap show the total number of enriched motifs/GO-terms, while the heat maps below show e) selected motifs or f) all GO-terms with “damage”, “repair”, “checkpoint”, “cell cycle” or “chromosome”, indicative of an activated DDR, respectively.

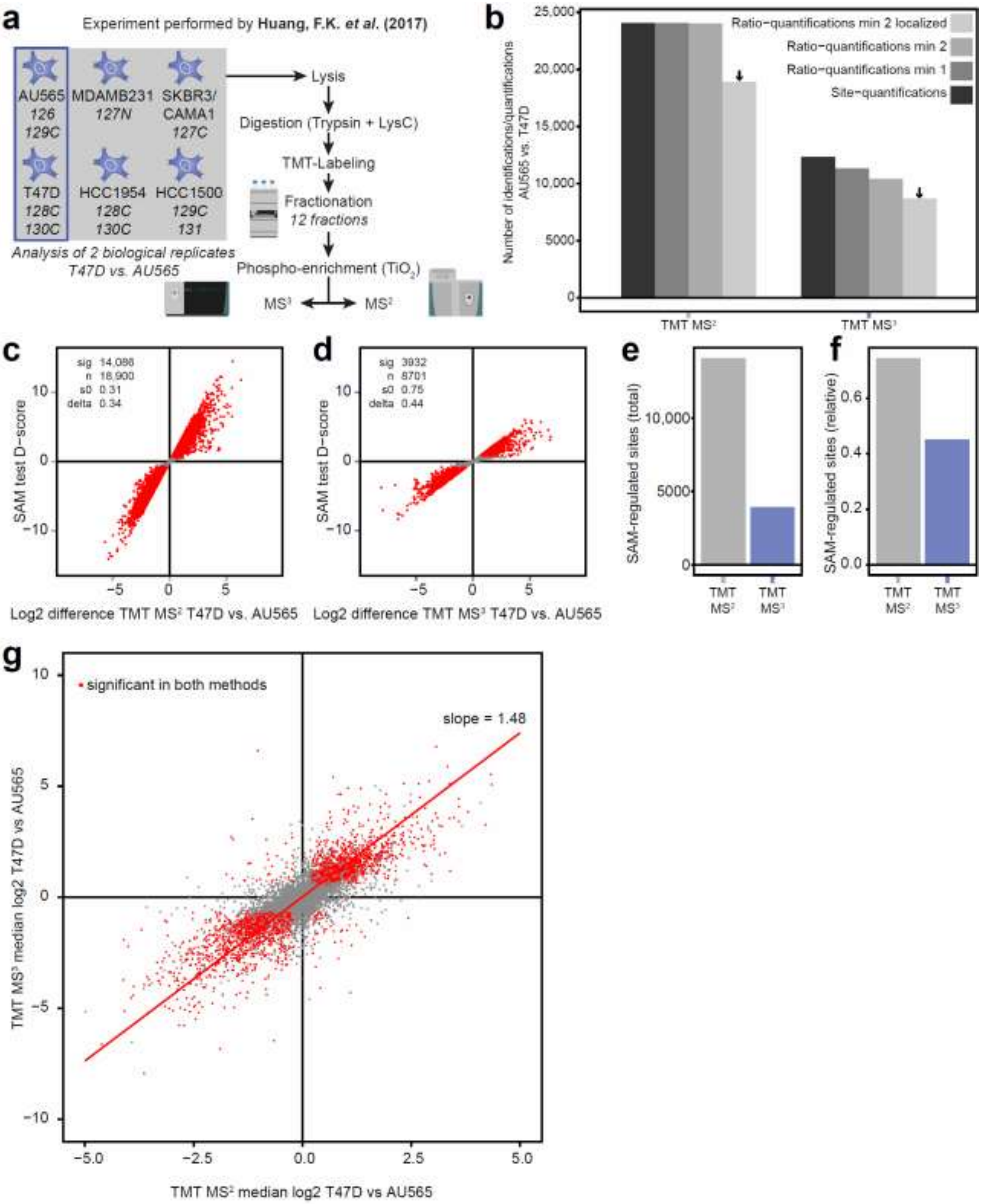
a
Experiment performed by Huang, F.K. et al. (2017)
AU565 126 129C
MDAMB231 127N
SKBR3/ CAMA1 127C
T47D 128C 130C
HCC1954 128C 130C
HCC1500 129C 131
Analysis of 2 biological replicates T47D vs. AU565
Lysis
Digestion (Trypsin + LysC)
TMT-Labeling
Fractionation 12 fractions
Phospho-enrichment (TiO2)
MS3
MS2
b
Number of identifications/quantifications AU565 vs. T47D
Ratio−quantifications min 2 localized
Ratio−quantifications min 2
Ratio−quantifications min 1
Site−quantifications
TMT MS2
TMT MS3
c
sig 14,086
n 18,900
s0 0.31
delta 0.34
SAM test D−score
Log2 difference TMT MS2 T47D vs. AU565
d
sig 3932
n 8701
s0 0.75
delta 0.44
SAM test D−score
Log2 difference TMT MS3 T47D vs. AU565
e
SAM-regulated sites (total)
f
SAM-regulated sites (relative)
g
significant in both methods
slope = 1.48
TMT MS3 median log2 T47D vs AU565
TMT MS2 median log2 T47D vs AU565

Supplementary Figure 5: Evaluation of quantification methods on a TMT data set published by Huang et al [2]. a) We reanalyzed a MS^2- and MS^3-measured TMT data set of different breast cancer cell lines, including AU565 and T47D measured as two technical replicates each. In their setup, Huang et al. fractionated peptides after TMT-labeling into twelve fractions on an Agilent 1100 system and subsequently enriched phosphopeptides from each fraction using titanium dioxide. Samples were then measured on an Orbitrap Fusion or Q Exactive HF, for MS^3- and MS^2-based TMT, respectively. The MS^3-setup they used roughly corresponds to the TMT MS^3 OT setup described in this manuscript. Raw files were downloaded from MassIVE and processed as described in the materials and methods section. b) Bar plot showing total numbers of quantified phosphopeptides for all replicates of each quantification method, respectively. Calculations of ratios were performed within biological replicates and filtered for measurement in a minimum of one or two replicates, and >75% confident phosphorylation site localization. For further analysis, ratios quantified in both replicates only and with a localization probability of at least 75% (black arrows) were used. c/d) SAM-based identification of significantly regulated phosphorylation sites was performed with two sample unpaired t-test and s0 estimation set to automatic for the c) TMT MS^2- and d) TMT MS^3-measured data. The FDR was decreased to 0.01 to better illustrate the relative differences between both quantification approaches. Significantly regulated phosphorylation sites (sig) are highlighted in red, non-significant ones in grey. Applied s0 and delta values, as well as the total number of tested phosphorylation sites (n) are shown. e/f) The bar plots show the number of significantly regulated phosphorylation sites for each quantification method e) in total, and f) as a fraction relative to the total number of tested sites. g) Correlation of MS^2-based and MS^3-based TMT quantified AU565 vs. T47D log2 ratios. The slope of the linear regression line of the SAM-regulated phosphorylation sites is shown and corresponds well to the value determined in this study for TMT MS^3 OT as shown in Fig 1 b).

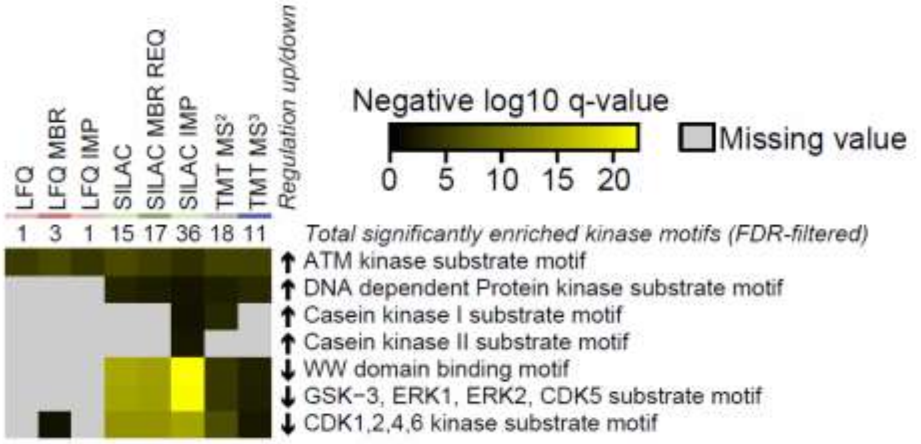

Supplementary Figure 6: Kinase motif enrichment of shared phosphorylation sites. This heat map shows kinase motif enrichments of significantly SAM-up/down-regulated phosphorylation sites from Fig. 4c) vs the respective non-regulated sites as background. Importantly, only sites quantified in all eight quantification approaches were considered, leading to a total of 1914 sites each. Enrichment was performed using Fisher exact tests within Perseus with relative enrichment on gene level and an FDR of 0.02. The numbers above the heat map show the total number of enriched motifs, while the heat maps below show selected motifs indicative of an activated DDR. Importantly, enrichment analysis of GO-terms did not yield significantly regulated terms after FDR-filtering for any of the eight quantification approaches.

Supplementary Tables

	(TMT) MS^2	TMT MS^3 OT	TMT MS^3 OT MC	TMT MS^3 IT NL	TMT MS^3 IT	(TMT) MS^2
MS instrument	Fusion Lumos	Fusion Lumos	Fusion Lumos	Fusion Lumos	Fusion Lumos	Q Exactive HF/HF-X
MS S-lens RF	30%	30%	30%	30%	30%	50%
MS spray voltage	2kV positive	2kV positive	2kV positive	2kV positive	2kV positive	2kV positive
MS capillary temp.	275°C	275°C	275°C	275°C	275°C	275°C
MS^1 readout	OT 120,000	OT 120,000	OT 120,000	OT 120,000	OT 120,000	OT 120,000
MS^1 scan range	400-1500	400-1500	380-1400	380-1400	380-1500	375-1500
MS^1 AGC target	4.00E+05	4.00E+05	4.00E+05	4.00E+05	2.00E+05	3.00E+06
MS^1 injection time	50msec	50msec	100msec	50msec	50msec	25msec
MS^1 dynamic exclusion	60sec	60sec	60sec	60sec	70sec	60msec
MS^2 readout	OT 60,000	OT 30,000	OT 30,000	IT Turbo	IT Turbo	OT 60,000
MS^2 first mass m/z	100					100
MS^2 AGC target	1.00E+05	5.00E+04	5.00E+04	1.00E+04	1.00E+04	1.00E+05
MS^2 injection time	110msec	60msec	60msec	50msec	50msec	110msec
MS^2 activation	HCD 30% vs. 38% (TMT)	CID 35% MSA*	CID 35% MSA*	CID 35%	CID 35% MSA*	HCD 28% vs. 33% (TMT)
MS^2 isolation width	0.8	0.7	0.7	0.7	0.7	0.8
MS^3 selection		SPS 10	SPS 10	NL targeted loss trigger 80 and*	SPS 10	
MS^3 readout		OT 60,000	OT 60,000	OT 60,000	OT 60,000	
MS^3 scan range		100-500	100-500	100-2000	120-500	
MS^3 AGC target		1.00E+05	1.00E+05	1.00E+05	1.00E+05	
MS^3 injection time		105msec	120msec	120msec	120msec	
MS^3 activation		HCD 65%	HCD 65%	HCD 40%	HCD 65%	
MS^1 for MS^3 isolation width		2	1.3/1.0/0.8/0.7 for z 2/3/4/5&6	2	2	
MS^2 for MS^3 isolation width				2		
topN	3sec	3sec	top40	3sec	3sec	top7

Supplementary Table 1: MS instrument settings. MS instrument methods used in Fig. 1-5 are shown, with non-applicable settings left blank. HCD = higher-energy collisional dissociation [1]; CID = collision-induced dissociation; MSA = multi-stage activation; NL = neutral loss; * NL mass 97.9673

Method	Ratio	total peptide ratios	yeast peptide ratios	human peptide ratios	s0	delta	significant peptides
LFQ	4v1	3401	136	3265	0.21	0.45	1040
LFQ	10v1	2890	133	2757	0.53	0.26	950
LFQ MBR	4v1	5716	327	5389	0.25	0.35	2265
LFQ MBR	10v1	4991	337	4654	0.55	0.25	1780
SILAC	4v1	1500	31	1469	3.12	0.12	170
SILAC	10v1	1500	31	1469	3.12	0.10	63
SILAC MBR REQ	4v1	2434	126	2308	3.33	0.10	498
SILAC MBR REQ	10v1	2434	126	2308	3.30	0.10	214
TMT MS^2	4v1	4335	789	3546	0.10	0.45	1321
TMT MS^2	10v1	4334	789	3545	0.08	0.45	2178
TMT MS^3 IT	4v1	2320	426	1894	0.02	0.50	734
TMT MS^3 IT	10v1	2320	426	1894	0.02	0.50	1235
TMT MS^3 OT	4v1	3154	539	2615	0.02	0.53	816
TMT MS^3 OT	10v1	3153	539	2614	0.03	0.40	2302
TMT MS^3 IT NL	4v1	1421	271	1150	0.02	0.52	331
TMT MS^3 IT NL	10v1	1421	272	1149	0.03	0.56	1094
TMT MS^3 OT MC	4v1	1911	319	1592	0.01	0.54	421
TMT MS^3 OT MC	10v1	1911	319	1592	0.03	0.58	1444
SILAC*	4v1	1500	31	1469	set 0.1	0.62	307
SILAC*	10v1	1500	31	1469	set 0.1	0.75	92
SILAC MBR REQ*	4v1	2434	126	1469	set 0.1	0.45	786
SILAC MBR REQ*	10v1	2434	126	1469	set 0.1	0.82	263

Supplementary Table 2: Technical benchmark SAM parameters. The table lists the total number of tested peptides for each quantification approach and ratio, and how many of the tested peptides were of yeast or human origin. SAM testing was performed using standard parameters, which means that s0 was calculated based on the data set at hand, and delta was estimated with an FDR cutoff of 20%. Please note that the numbers of peptides deemed significant by the SAM test are only valid for the calculated delta cutoff. This delta cutoff was not used in the ROC plot, which instead was calculated directly using the calculated d-scores. The s0 values for SILAC are very high compared to LFQ and SILAC, which is most likely caused by the inherent biological variation of this setup for SILAC, and our inability to apply normalization strategies between ratio channels without affecting the quantification accuracy of the yeast peptides. Nevertheless, we repeated SAM-testing with fixed s0 values of 0.1 for SILAC (marked by *), and this actually slightly decreases its performance in the ROC analysis, as shown in Supplementary Fig. 2b).

Statistical test	LFQ	LFQ MBR	LFQ IMP	SILAC	SILAC MBR REQ	SILAC IMP	TMT MS^2	TMT MS^3
Total sites tested	3254	5143	9420	4453	5098	9420	21,563	13,640
SAMR paired t-test	62	279	738	908	5045	2140	8783	9420
SAMR row-norm. + unpaired t-test	2146	3404	598	3962	49	8862	15,505	9897
Perseus paired t-test s0 = 0.1	0	113	0	2011	2370	2502	2999	1353
Perseus paired t-test s0 = 0.2	0	124	0	1836	2094	2211	2961	1483
Perseus row-norm. + unpaired t-test s0 = 0.1	168	389	20	2721	123	4091	5877	3167
Perseus row-norm. + unpaired t-test s0 = 0.2	155	422	20	2314	117	2997	5180	3126

Supplementary Table 3: Evaluation of quantification methods with different statistical approaches. The table lists the total number of tested phosphorylation sites for each quantification approach, and how how many of them were deemed statistically significantly regulated by different test settings and normalization approaches. SAM testing was performed using standard parameters (s0 determination automatic, delta estimation with an FDR cutoff of 0.20). Perseus FDR-corrected t-testing was performed using standard settings (FDR = 0.05), with s0 set to either 0.1 (Perseus-default) or 0.2, which were found to be common choices in the literature. In addition to the paired t-test, which was used to correct for day-to-day variance in the biological replicates, a different normalization approach was used together with unpaired t-testing. In this approach, both conditions DOX and C of each biological replicate for each method were normalized by subtracting the average between them in log2 space (= row normalization). Similarly to the paired t-test, this should assure that the statistical test detects biological changes caused by the treatment, and not the day-to-day variation in cell culture and lysing procedure.

Supplementary Notes

Supplementary Note 1: Calculation of absolute phosphorylation site stoichiometry via a 3D multiple regression model. This is a description of the theoretical reasoning for the 3DMM phosphorylation site stoichiometry calculation.

In order to calculate absolute phosphorylation site stoichiometry, TMT reporter ion intensities (or other normalized/multiplexed intensities) of singly-phosphorylated peptides (phos), their respective non-phosphorylated peptide-variants (non), and their respective proteins (protein) are needed.

With *N* as the total number of peptides, we expect for singly-phosphorylated peptides that:

$$N^{\mathrm{phos}} + N^{\mathrm{non}} = N^{\mathrm{protein}} \; (1)$$

Furthermore, we can relate the number of peptides *N* with the intensity of peptides *I* via their respective peptide “flyabilities” *f*:

$$N^{\mathrm{phos}} * f^{\mathrm{phos}} = I^{\mathrm{phos}} \; (2)$$

$$N^{\mathrm{non}} * f^{\mathrm{non}} = I^{\mathrm{non}} \; (3)$$

$$N^{\mathrm{protein}} * f^{\mathrm{protein}} = I^{\mathrm{protein}} \; (4)$$

We can subset 2-4 in 1 and receive:

$$\frac{I^{\mathrm{phos}}}{f^{\mathrm{phos}}} = \frac{I^{\mathrm{protein}}}{f^{\mathrm{protein}}} - \frac{I^{\mathrm{non}}}{f^{\mathrm{non}}}$$

$$I^{\mathrm{phos}} = I^{\mathrm{protein}} * \frac{f^{\mathrm{phos}}}{f^{\mathrm{protein}}} - I^{\mathrm{non}} * \frac{f^{\mathrm{phos}}}{f^{\mathrm{non}}}$$

$$I^{\mathrm{phos}} = I^{\mathrm{protein}} * m_1 - I^{\mathrm{non}} * m_2 \; (5)$$

Thus, we apply multiple regression for these three intensities over different conditions. We can then extract one slope m_2 for all conditions, which we use to calculate the condition-specific factor *a* for each individual condition cond:

$$a_{\mathrm{cond}} = \frac{N^{\mathrm{phos}_{\mathrm{cond}}}}{N^{\mathrm{non}_{\mathrm{cond}}}} = \frac{I^{\mathrm{phos}_{\mathrm{cond}}}}{I^{\mathrm{non}_{\mathrm{cond}}}} * \frac{f^{\mathrm{non}}}{f^{\mathrm{phos}}} = \frac{I^{\mathrm{phos}_{\mathrm{cond}}}}{I^{\mathrm{non}_{\mathrm{cond}}}} * \frac{1}{m_2} \; (6)$$

We can now calculate the occupancy of a given phosphorylation site for a given condition via:

$$\mathrm{Occupancy}_{\mathrm{cond}} = \frac{N^{\mathrm{phos}_{\mathrm{cond}}}}{N^{\mathrm{phos}_{\mathrm{cond}}} + N^{\mathrm{non}_{\mathrm{cond}}}} = \frac{\frac{N^{\mathrm{phos}_{\mathrm{cond}}}}{N^{\mathrm{non}_{\mathrm{cond}}}}}{\frac{N^{\mathrm{phos}_{\mathrm{cond}}}}{N^{\mathrm{non}_{\mathrm{cond}}}} + 1} = \frac{a_{\mathrm{cond}}}{a_{\mathrm{cond}} + 1} \; (7)$$

Supplementary References

1. Olsen, J. V. *et al.* Higher-energy C-trap dissociation for peptide modification analysis. *Nat. Methods* **4,** 709–712 (2007).
2. Huang, F.-K. *et al.* Deep Coverage of Global Protein Expression and Phosphorylation in Breast Tumor Cell Lines Using TMT 10-plex Isobaric Labeling. *J. Proteome Res.* **16,** 1121–1132 (2017).
