# Supplementary material for: Benchmarking common quantification strategies for large-scale phosphoproteomics
Source: Nat Commun. 2018 Mar 13;9:1045. doi: 10.1038/s41467-018-03309-6 (PMC5849679; doi:10.1038/s41467-018-03309-6)
Supplement: Supplementary file 3 — Description of Additional Supplementary Files [file 41467_2018_3309_MOESM3_ESM.pdf]

Description of Additional Supplementary Files

File Name: Supplementary Data 1
Description: **R code for phosphorylation stoichiometry calculation via 3DMM.** This R script file contains the R code needed to perform the 3DMM phosphorylation stoichiometry calculation for Fig. 6.

File Name: Supplementary Data 2
Description: **Example data for 3DMM in Fig. 6.** This compressed file contains the modificationspecificpeptides.txt output from MaxQuant and the HumanAndYeastProtIdentifiers.txt to differentiate between human and yeast proteins, both of which are needed to recreate the analysis in Fig. 6 with the R code in Supplementary Data 1.

File Name: Supplementary Data 3
Description: **Overview of MS raw files and MaxQuant configuration settings.** This xlsx-file features an overview of all MS raw files, their association with the Figures and MaxQuant configuration settings.

File Name: Supplementary Data 4
Description: **Tools for signal-to-noise extraction in Fig. 1.** This Perl script file contains the code to read out TMT reporter ion signal-to-noise ratios from raw data after processing using raxport.exe.

File Name: Supplementary Data 5
Description: **Biological benchmark phosphorylation site intensities and SAM regulation.** This xslx-file features all intensities and SAM-test statistics of the biological benchmark dataset in Fig. 3.

File Name: Supplementary Data 6
Description: **Kinase motif and GO-term enrichment data.** This xlsx-file features all enriched kinase motifs and GO-terms from Fig. 4 and Supplementary Fig. 4/6.
